# A Novel Lipopeptide from Skin Commensal Activates TLR2/CD36-p38 MAPK Signaling to Increase Antibacterial Defense against Bacterial Infection

**DOI:** 10.1371/journal.pone.0058288

**Published:** 2013-03-05

**Authors:** Dongqing Li, Hu Lei, Zhiheng Li, Hongquan Li, Yue Wang, Yuping Lai

**Affiliations:** Shanghai Key Laboratory of Regulatory Biology, School of Life Sciences, East China Normal University, Shanghai, People’s Republic of China; French National Centre for Scientific Research, France

## Abstract

*Staphylococcus epidermidis* (*S.epidermidis*) plays important protective roles by directly producing or by stimulating hosts to produce antimicrobial peptides (AMPs) against pathogenic infections. Although several AMPs from *S.epidermidis* have been identified, molecules that stimulate hosts to produce AMPs remain largly unknown. Here we demonstrate that a new lipopeptide (named LP01) purified from *S.epidermidis* culture media has a unique structure with heneicosanoic acid (21 carbons) binding to lysine^11^ of a peptide chain. *In vitro* LP01 increased the expression of β-defensin 2(hBD2) and hBD3 in neonatal human epidermal keratinocytes(NHEK), leading to increased capacity of cell lysates to inhibit the growth of *S.aureus*. *In vivo* LP01 induced the expression of mouse β-defensin 4(mBD4) to decrease the survival of local *S.aureus* in skin and systemic *S.aureus* survival in liver. The induction of beta-defensins by LP01 was dependent on TLR2 as *Tlr2*-deficient mice had decreased mBD4. Furthermore, knockdown of CD36 decreased the expression of hBD2 and hBD3, and p38 MAPK inhibitor significantly inhibited the expression of hBDs induced by LP01.Taken together, these findings demonstrate that lipopeptide LP01 from normal commensal *S.epidermidis* increases antimicrobial peptide hBD2 and hBD3 expression via the activation of TLR2/CD36-p38 MAPK, thus enhancing antimicrobial defense against pathogenic infections.

## Introduction

Skin, as a first line of defense, interfaces with the environment and is exposed to a myriad of microbes. Among these microbes, *Staphylococcus epidermidis* (*S.epidermidis*) is the most abundant bacterium that resides on skin and generally has a benign relationship with its host. Previously, we have gained considerable insights into beneficial roles of *S.epidermidis* by balancing inflammatory responses after skin injury. We discovered that lipoteichoic acid from *S.epidermidis* substantially attenuated keratinocyte response to skin injury through a TLR2-dependent inhibition of the TLR3 signaling via TNF receptor-associated factor 1 (TRAF1), thus suppressing unwanted inflammatory cytokine production [1]. Besides the regulation of inflammation in skin injury, other groups showed that *S.epidermidis* produced some antimicrobial molecules including Staphylococcin 1580 [2], Pep5 [3], PSMs [4] to benefit cutaneous immune defense by selectively inhibiting the survival of skin pathogens. We have also demonstrated that less than 10 kDa molecules from *S.epidermidis* culture media induced the production of β-defensins to enable the skin to mount an enhanced response to pathogens [5]. However, the identity of the molecule from *S.epidermidis* to induce β-defensins remains unknown.

Besides *S.epidermidis,* several other bacteria have been shown to produce lipopeptides to help hosts against pathogenic infections by disturbing gram-positive bacterial cell wall synthesis [6]. One of the most well-established lipopeptides with direct antimicrobial activity is daptomycin [6]. This lipopeptide can destruct the membrane of Gram-positive pathogens including Methicillin-resistant *Staphylococcus aureus*. In addition to daptomycin, other lipopeptides including Iturin A [7] and Fengycin [8] from *Bacillus subtilis*, polymyxins from *Paenibacillus polymyxa* [9] and fusaricidins from *Paenibacillus polymyxa* [10] have been identified to exert their direct antimicrobial activity. Besides direct antimicrobial activity, synthetic lipopeptides such as MALP-2 can induce antimicrobial peptide expression in several cell types such as keratinocytes [5]. These observations thereby raise the possibility that the molecule from *S.epidermidis* to induce AMP might be a lipopeptide.

Given that skin commensal bacteria increase host defense against pathogenic infections and lipopeptides from bacteria exert direct antimicrobial activity or induce host to produce antimicrobial peptides, we hypothesized that the molecule from *S.epidermidis* to induce β-defensins might be a kind of lipopeptide. In this study we successfully purified one novel lipopeptide from *S.epidermidis* culture media and further delineated the mechanism by which the lipopeptide induced AMP against *S.aureus* infection. Our findings reveal the potential use of commensal bacterium-derived lipopeptides in treatment of skin infections.

## Materials and Methods

### Bacterial Strains and Mice

*Staphylococcus epidermidis* 1457, *Staphylococcus aureus*, *Escherichia coli DH5a* and *Propionibacterium acnes* were stored in our laboratory. *S.epidermidis* 1457 and *S.aureus* were cultured at 37°C for 16 h in Tryptic Soy Broth (TSB) medium (Sigma, St Louis, MO). *E.coli DH5a* was cultured at 37°C for 16 h in Lysogeny Broth (LB) medium. *P.acnes* was culture at 37°C in Reinforced Clostridial Medium in anaerobic pouch (MGC, JAPAN). All mice were kept under specific pathogen free conditions and maintained in accordance with the institutional guidelines. All animal experiments were approved by East China Normal University Animal Care and Use Committee.

### Lipopeptide Purification and Identification

*S.epidermidis 1457* was grown in TSB medium at 37°C for overnight. Next day, the overnight culture was diluted 1∶100 into TSB and grown for another 16 hours. Bacterial supernatants were collected and filtered by 0.22 µm stericup. Bacterium-free culture supernatant was adjusted to pH = 2.0 and then stored at 4°C for overnight. Next day, the crude lipopeptide was extracted by methanol at 37°C for overnight and dried by rotary evaporation followed by loading on HPLC with a C18 column for further purification. The purified lipopeptide was analyzed by thin-layer chromatography (TLC) on silica-coated glass plates in solvent system (butyl alcohol: acetic acid: H2O = 4∶2:1, v/v/v). Protein sequences were analyzed by Q-TOF MS/MS *de-novo* sequencing (Waters, ACQUITY^TM^ UPLC&Q-TOF Premier). The structure of lipid of the lipopeptide was analyzed by GC/MS (Shimadzu, GC/MS-QP2010).

### Real-time Quantitative RT-PCR

Total RNA was prepared using Trizol Reagent (TaKaRa, Japan) following the manufacturer’s instructions. RNA was quantified by Thermo NANODROP 2000 spectrophotometer. Total RNA (1 µg) was reverse transcribed using PrimeScript^®^ RT reagent Kit (TaKaRa, Japan) according to the manufacturer’s instructions. Real-time RT-PCR was conducted on Mx3005P (Stratagene, USA) using SYBR^®^ Premix ExTaq (TaKaRa, Japan). The quantification of gene expression was determined by the comparative 2ΔΔ*C_T_* method. The primers used in this manuscriptare shown in the following: mBD4 forward: GGCTTCAGTCAT GAGGATCCAT; mBD4 reverse: TTTGGGTAAAGGCTGCAAGTG; mBD14 forward: GTGGC CGGTGTGCTGTACT; mBD14 reverse: CGCTATTAGA ACATCGACCTATTTGT; hBD2 forward: CCAGCCATCAGCCATGAGGGT; hBD2 reverse: GGAGCCCTTTCTGAATCCGCA; hBD3 forward: GCCTCTT CCAGGTGTTTTTG; hBD3 reverse: GAGACCACAGGTGCCAATTT. The relative expression levels were determined by normalizing expression to 18s rRNA or glyceraldehyde 3-phosphate dehydrogenase (GAPDH). All the assays were performed in triplicate and repeated at least two times.

### Primary Cell Culture and Stimulation by Lipopeptide

Neonatal human epidermal keratinocytes (Cascade Biologics, USA) were cultured in EpiLife medium supplemented with EDGS, 0.06 mM CaCl_2_ (Cascade Biologics, USA) and Pen Strep(100 units/ml Penicillin and 100 µg/ml Sreptomycin). Murine primary keratinocytes were isolated from newborn skin by using dispase II (Sigma, St Louis, MO) and cultured in 154CF medium supplemented with HKGS and 0.2 mM CaCl_2_ and Pen Strep(100 units/ml Penicillin and 100 µg/ml Sreptomycin) (Invitrogen, Shanghai). Keratinocytes were seeded in 6-, 12- or 24-well plates to grow to 70% confluence. To test whether the induction of hBDs by lipopeptide was in a dose-dependent manner, 2.5, 5, 10, 15, 20, 25, 30 µg/ml lipopeptide was used to stimulate NHEK cells. 15 µg/ml lipopeptide was used to stimulate murine primary keratinocytes. 24 h later, cells were harvested. The expression of genes was analyzed by using real-time RT-PCR and the protein levels of hBDs were determined by hBD2 and hBD3 ELISA Kit (Peprotech, Hamburg, Germany).

### Bacterial Killing Assay

To test the capacity of NHEK cell lysate stimulated with LP01 in the inhibition of the growth of bacteria, NHEK cells were cultured in no P/S Epilife medium and then were treated with 15 µg/mL of LP01. After 24 hours, 100 µL of phosphate buffered saline (PBS) containing protease inhibitor cocktail was added into each well. Cells were collected by cell scraper and then sonicated on ice-cold water. After removed cell debris by centrifugation, the concentration of the lysates was determined by BCA^TM^ Protein Assay Kit (Novagen, San Diego, CA). 10 µg of cell lysates was incubated with 1×10^6^ CFU *P.acnes*, *S.aureus* or *S.epidermidis* at 37°C for 3 h. The bacterial were then serially diluted by 10-fold with PBS and plated on RCM agar plates (*P.acnes*), TSB agar plates (*S.aureus, S.epidermidis*) or LB agar plates (*E.coli DH5a*).

### *S.aureus* Infection *in vivo*

The backs of 8-week adult mice were shaved and hair was removed by chemical depilation. 2 mg/kg of LP01 and PBS was intradermally injected into mouse back, respectively. Next day, 50 µL of live *S.aureus* (OD_600_ = 0.7–0.8) complexed with cytodex beads (Sigma, St Louis, MO) as carrier was intradermally injected 2 hours after injection of LP01. Lesional sizes caused by *S.aureus* infection were measured daily. At day 3, skin around the lesional sites, liver and spleen were taken and homogenized for *S.aureus* culture.

### Inactivation of TLR2 or p38 MAPK

TLR2 inhibitor OxPAPC (InvivoGen, San Diego, CA) or p38 MAPK inhibitor SB202190(Sigma, St Louis, MO) was added 10 minutes before normal human keratinocytes or murine primary keratinocytes were treated with 15 µg/ml of LP01. 24 hours later, total RNA was extracted and cDNA synthesized by the PrimeScript^®^ RT reagent Kit (TaKaRa, Japan). The level of gene expression was quantified by real-time RT-PCR by using Stratagene Mx3005P.

### Immunoblotting

Normal human epidermal keratinocytes were treated with 15 µg/mL of LP01 for 1 hour in the presence or absence of inhibitors. Cells were lysed in the RIPA buffer (pH 7.4) containing protease inhibitor cocktail (Roche, Pleasanton, CA) after washed by ice-cold PBS for 3 times. Protein concentrations of the extracts were measured by BCA^TM^ Protein Assay Kit (Novagen, San Diego, CA). 30 µg of total protein was used for western blot. The lysates were separated by 12% SDS-PAGE and analyzed by immunoblotting with phosphorylated p38 MAPK antibody or p38 MAPK antibody (Cell signaling, Danvers, MA), respectively. The membrane was scanned by Odyssey machine (Li-Cor Biosciences, Lincoln, NE).

### Statistical Analysis

All data are present as mean±SEM. Two-tailed t-test was used to determine significances between two groups. The significances among multiple groups were determined by One-way or Two-way ANOVA with Bonferroni post test of GraphPad Prism Version 5 (San Diego, CA). For all statistical tests, we considered *P* values <0.05 to be statistically significant.

## Results

### The Structure of Lipopeptide 01 (LP01) from Skin Commensal *Staphylococcus epidermidis*

**Figure 1:**
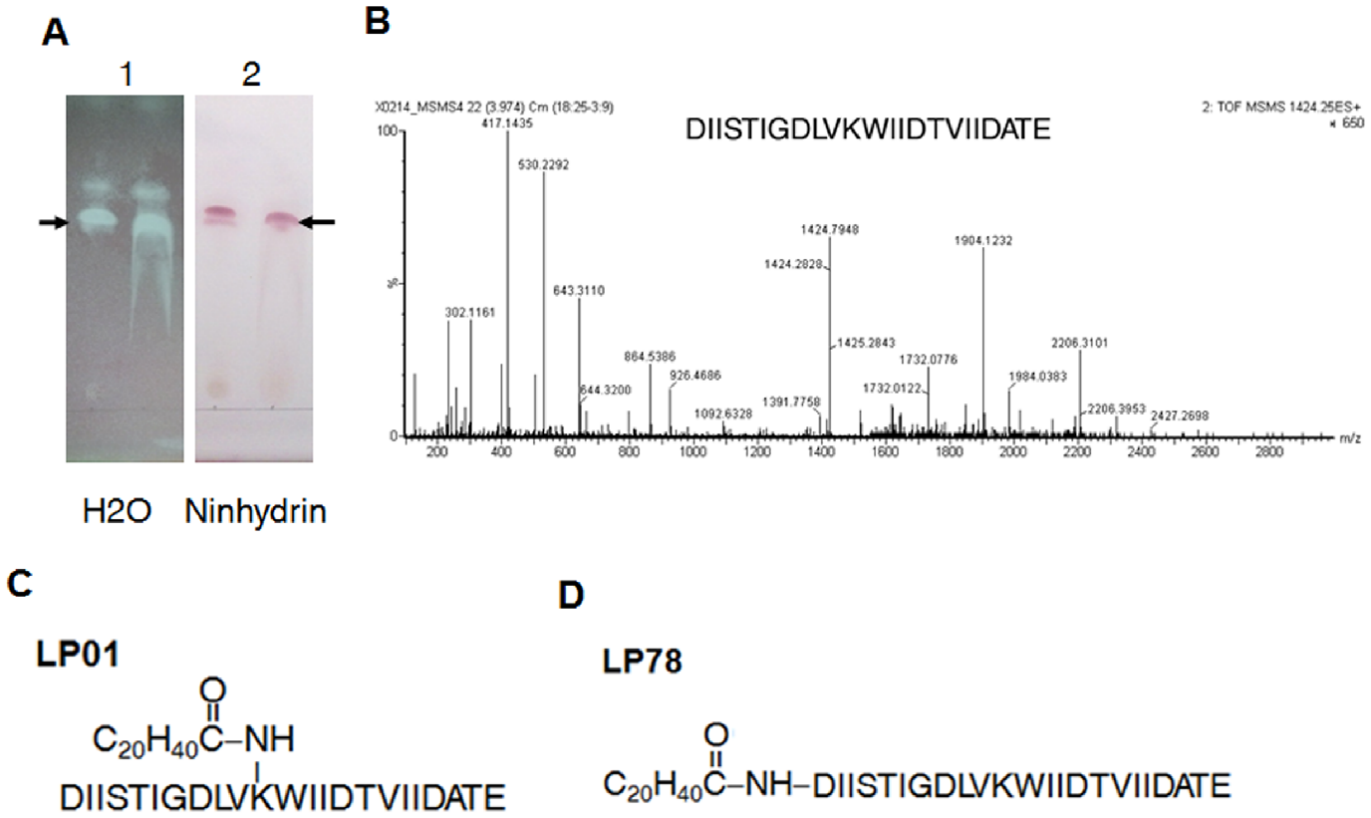
The identification of the lipopeptide from *Staphylococcus epidermidis.* **A.** The analysis of the lipopeptide from *S.epidermidis* by thin-layer chromatography. Duplicate samples were loaded on TLC plate and molecules with different hydrophobicity were separated. Water was used to show hydrophobicity of lipopeptides and ninhydrin was used to show that the lipopeptide contains amino acids. The arrows indicate LP01. **B.** Q-TOF MS/MS analysis of lipopeptide in positive-ion model. Q-TOF MS/MS analysis showed that the amino acid sequence of the lipopeptide was DIISTIGDLVKWIIDTVIIDATE. **C&D.** Two possible structures of the lipopeptide. Aspartic acid (D^1^) at N-terminus and lysine (K^11^) are two amino acids with free NH_3_^+^, and free -COOH of heneicosanoic acid might react with NH_3_^+^ to form CO–NH. Thereby heneicosanoic acid may bind to ^1^D or ^11^K of the peptide chain.

We have shown that less than 10 kDa molecules from *S.epidermidis* culture media induced the production of β-defensins and that one synthetic lipopeptide such as MALP-2 induced hBDs expression in keratinocytes [5], we thus hypothesized that the molecule from *Staphylococcus epidermidis* to induce β-defensins might be a lipopetide. To test this, we used methanol extraction, acid precipitation, thin-layer chromatography and high-performance liquid chromatography (HPLC) to isolate lipopeptides from the *S.epidermidis* culture media(Fig. 1) and confirmed that the lipopeptide we purified from *S.epidermidis* culture media had the capacity to induce huaman beta-defensin 2 (hBD2) and hBD3 expression in neonatal human epidermal keratinocytes (NHEKs)(data not shown). The *de-novo* peptide sequencing showed that the peptide chain of the lipopeptide is DIISTIGDLVKWIIDTVIIDATE(Fig. 1B), and Gas chromatography mass spectrometry revealed that this lipopeptide contained one heneicosanoic acid C_20_H_40_COOH(Fig. 1C and 1D). The analysis of the peptide sequence showed that aspartic acid (D^1^) at N-terminus and the amino acid at site 11 lysine (K^11^) might be amino acids where heneicosanoic acid bound to the peptide chain. Therefore, structures of the lipopeptide might be as shown in Fig. 1C and Fig. 1D.

**Figure 2:**
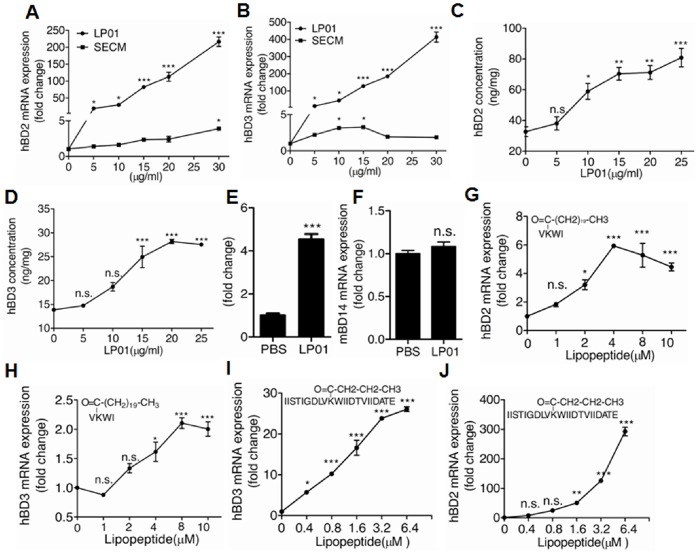
LP01 induces β-defensin expression in primary keratinocytes. A&B. Quantification of hBD2 and hBD3 mRNA expression in NHEKs treated with LP01 or SECM. **C&D.** hBD2 and hBD3 protein expression in cell lysates of NHEKs treated with LP01 by ELISA. **E&F.** Quantification of mBD4 and mBD14 mRNA expression stimulated with 15 µg/mL LP01 in primary murine keratinocytes. Primary murine keratinocytes were isolated from C57BL/6 mice. **G&H.** Quantification of hBD2 and hBD3 mRNA expression in NHEKs stimulated by LP01 with shortened peptide chain. **I&J.** Quantification of hBD2 and hBD3 mRNA expression in NHEKs stimulated by LP01 with shortened fatty acid chain. *P<0.05, **P<0.01 and ***P<0.001, n.s., no significance. *P* values were determined by one-way ANOVA or two-tailed t test. Data are the means ± SEM of *n* = 3 and representative of two independent experiments.

To determine the structure-function relationship of the lipopetides, we synthesized the above two possible lipopeptides LP01 and LP78 and then evaluated their capacity to induce the expression of antimicrobial peptides. Compared to *S.epidermidis* culture media (SECM), synthetic LP01 significantly increased both mRNA and protein of hBD2 and hBD3 in NHEKs (Fig. 2A–2D) while synthetic LP78 did not (data not shown). In addition to NHEKs, LP01 significantly increased the expression of mouse beta-defensin 4 (mBD4, mouse homolog of human hBD2) but not mBD14 (mouse homolog of human hBD3) in primary murine keratinocytes (Fig. 2E and 2F). Furthermore, the capacity of LP01 to induce hBDs was more dependent on the intact peptide chain than the intact fatty acid. This was made evident by the fact that a shortened peptide chain with heneicosanoic acid markedly decreased hBDs induction while the intact peptide chain with shortened fatty acid slightly decreased hBDs induction (Fig. 2 G–J). These data suggest that LP01 with heneicosanoic acid binding to peptide chain (22 amino acids) is the lipopeptide from *S.epidermidis* to induce beta-defensins.

### LP01 Increases Antibacterial Activity against *S.aureus* Infection

**Figure 3:**
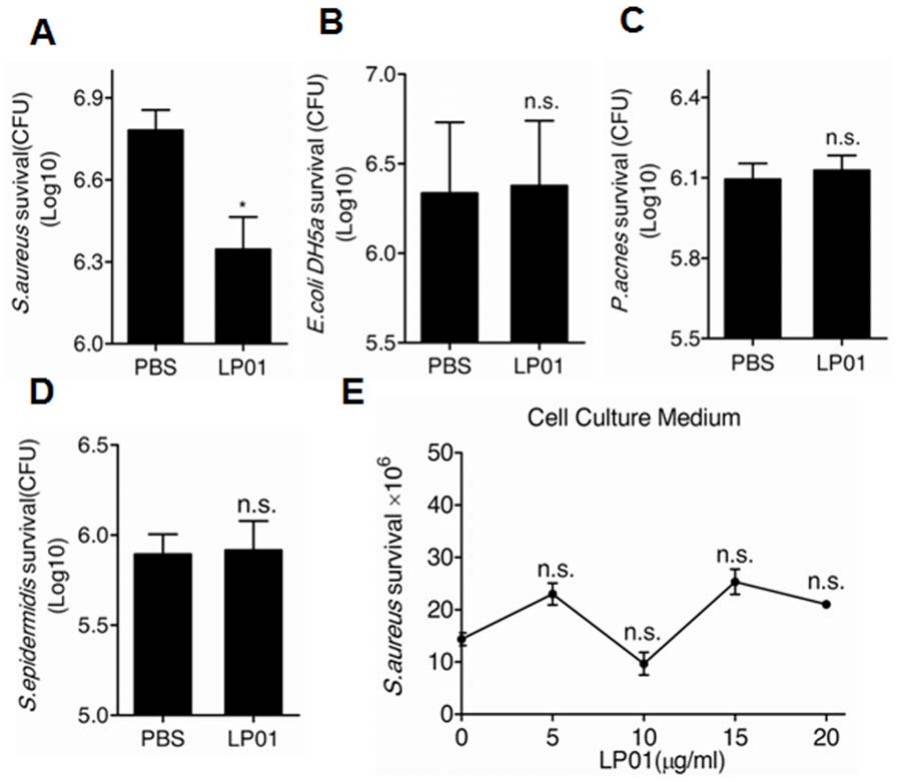
LP01 increases antibacterial activity of keratinocytes. The growth of *S.aureus* (**A**), *E.coli DH5a* (**B**), *P.acnes* (**C**) and *S.epidermidis* (**D**) after exposure to lysates of LP01-treated neonatal human epidermal keratinocytes or the growth of *S.aureus* after exposure to cell culture medium of LP01-treated NHEKs (**E**). The bacterial were serially diluted 10-fold with PBS and then counted by colony formation. *P<0.05; n.s., no significance. *P* values were determined by t-test. All data are representative of three independent experiments with *n* = 3 and are means ± SEM.

It is known that human hBD2 and hBD3 from epidermal keratinocytes exert bactericidal activity against *E.coli* and *S.aureus* infection [11], [12]. Since LP01 significantly induced hBD2 and hBD3 protein expression in kerationcytes (Fig. 2A–2D) but not too much in cell culture media (data not shown), we next examined whether the induction of hBD2 and hBD3 by LP01 could increase the antibacterial capacity of keratinocytes *in vitro*. A cell lysate of undifferentiated NHEKs pretreated with 10 µg/ml of LP01 significantly inhibited the growth of *S.aureus* (Fig. 3A), but did not inhibit *E.coli* (Fig. 3B), *P.acnes* (Fig. 3C), and *S.epidermidis* itself (Fig. 3D). Moreover, due to the low secreted hBD2 and hBD3, cell culture media of NHEKs treated with LP01 was not able to inhibit the growth of *S.aureus* (Fig. 3E).

**Figure 4:**
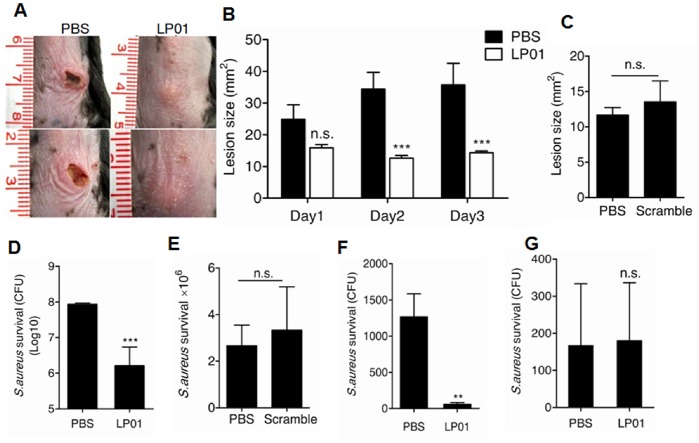
LP01 protects mice from *S.aureus* infection. **A.** Photograph of skin lesions caused by *S.aureus* at 3 days after *S.aureus* injection. **B.** ImageJ analysis of the lesional size of A. **C.** ImageJ analysis of the lesional size of PBS- or scrambled lipopeptide-treated mice. Local *S.aureus* survival in skin (**D and E**) and systemic *S.aureus* survival in liver (**F**) and spleen (**G**) of PBS- or scrambled lipopeptide- or LP01- pretreated mice. **P<0.01; ***P<0.001. *P* values were determined by two-tailed t test or two-way ANOVA. All data are the means ± SEM of *n* = 6 and representative of two independent experiments.

To confirm that the induction of antimicrobial peptides in keratinocytes by LP01 would be relevant to protection against *S.aureus* infection *in vivo*, LP01 was intradermally injected into mice 24 and 2 hours before an infectious challenge at the site with *S.aureus*. LP01-treated mice showed significantly smaller infectious skin lesions when compared with control mice injected with PBS or scrambled lipopeptide (Fig. 4A–4C). Accompanied with smaller infectious lesions, the survival of *S.aureus* at the local site of infection was significantly decreased (Fig. 4D and 4E). In addition, the survival of *S.aureus* in liver, but not in spleen, was decreased (Fig. 4F and 4G). Taken together, these results demonstrate that the LP01 from *S. epidermidis* increases host defense aganist *S. aureus* infection.

### The Induction of Beta-defensins by LP01 is Dependent on TLR2 and CD36

**Figure 5:**
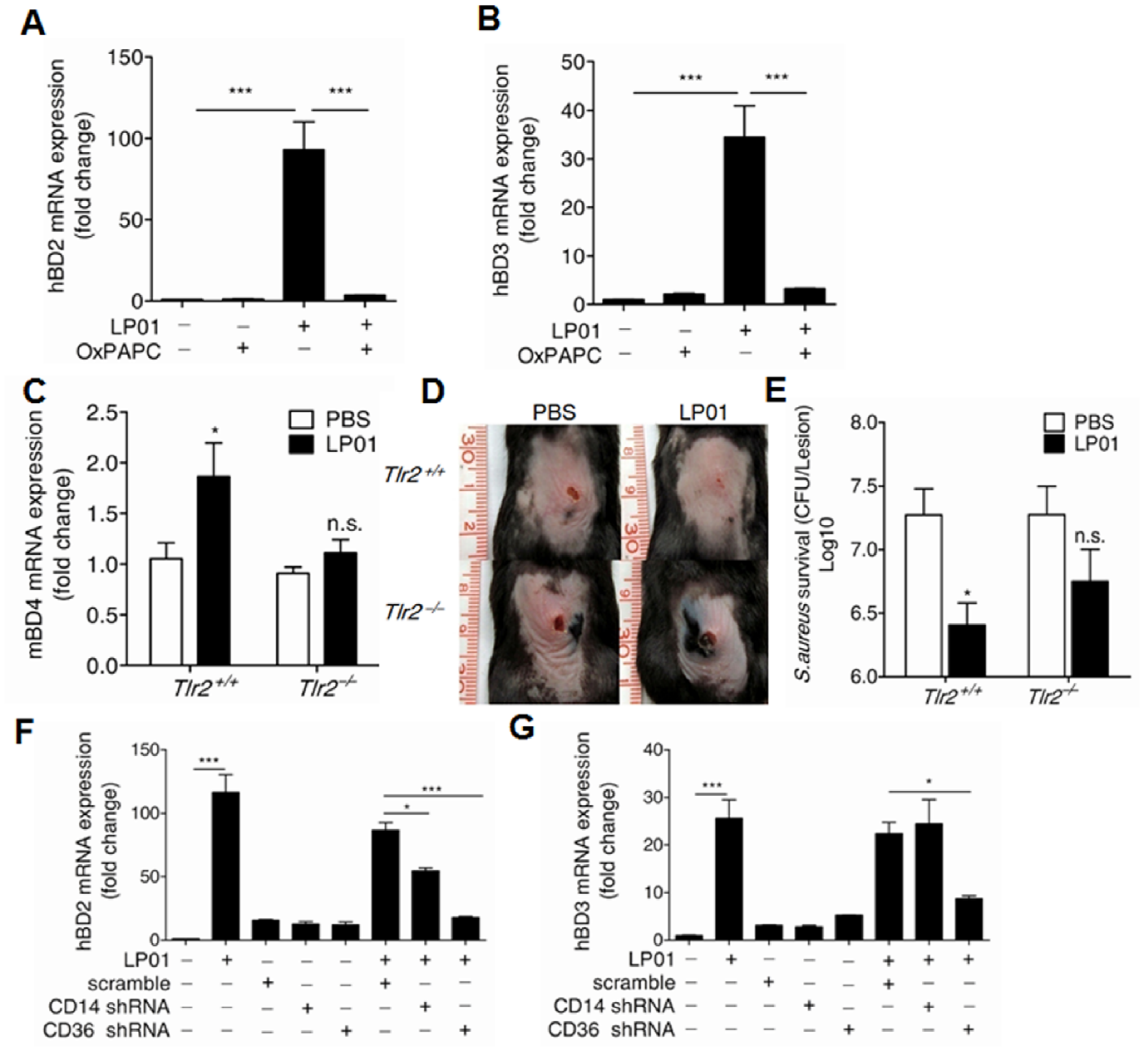
The induction of beta-defensins by LP01 is dependent on TLR2. **A, B.** The expression of hBD2 and hBD3 in NHEKs treated with 15 µg/mL of LP01 in the presence or absence of TLR2 inhibitor OxPAPC. **C.** mBD4 expression in mouse ears 24 h after injection by 2 mg/kg of LP01. **D.** Photograph of skin lesions caused by *S.aureus* at 3 days after *S.aureus* injection in *Tlr2^+/+^* and *Tlr2^–/–^* mice. **E.** *S.aureus* survival in skin of PBS- and LP01- pretreated *Tlr2^+/+^* and *Tlr2^–/–^* mice. **F–G.** Quantification of hBD2 and hBD3 expression in NHEK cells stimulated with LP01 after CD14 or CD36 was silenced. *P<0.05, ***P<0.001. *P* values were determined by one-way or two-way ANOVA. All data are representative of two independent experiments with *n* = 3–6 and are the means ± SEM.

Having established the role of LP01 in *S.aureus* skin infection, we next sought to explore the mechanism by which LP01 regulates antimicrobial peptide expression. Since TLR2 is a well-known receptor on keratinocytes for lipopeptides [13], [14], we assumed that the LP01 isolated from *S.epidrmidis* would activate TLR2 to induce antimicrobial peptide expression. To test this, we first used the TLR2 inhibitor OxPAPC to block TLR2 activation. OxPAPC completely inhibited the expression of hBD2 and hBD3 induced by LP01 (Fig. 5A and 5B). Consistent with our *in vitro* observations, LP01 lost its capacity to induce the expression of mBD4 in *Tlr2*-deficient mice compared to wild-type mice (Fig. 5C). In addition, LP01 failed to protect mice from *S.aureus* infection in *Tlr2*-deficient mice as shown with bigger infectious skin lesions and increased surviving *S.aureus* (Fig. 5D and 5E). Furthermore, knockdown of a coreceptor for TLR2 heterodimer, CD36, significanlty decreased hBD2 and hBD3 expression induced by LP01 (Fig. 5F and 5G). However, knockdown of antoher coreceptor for TLR2 heterodimer, CD14, only significantly decreased LP01-induced hBD2 expression, but not hBD3 expression (Fig. 5F and 5G). Altogether, these data demonstrate that lipopeptide LP01 activates TLR2 and CD36 to induce hBD2 and hBD3 in skin.

### The Activation of p38 MAPK is Required for the Induction of Beta-defensins by LP01

**Figure 6:**
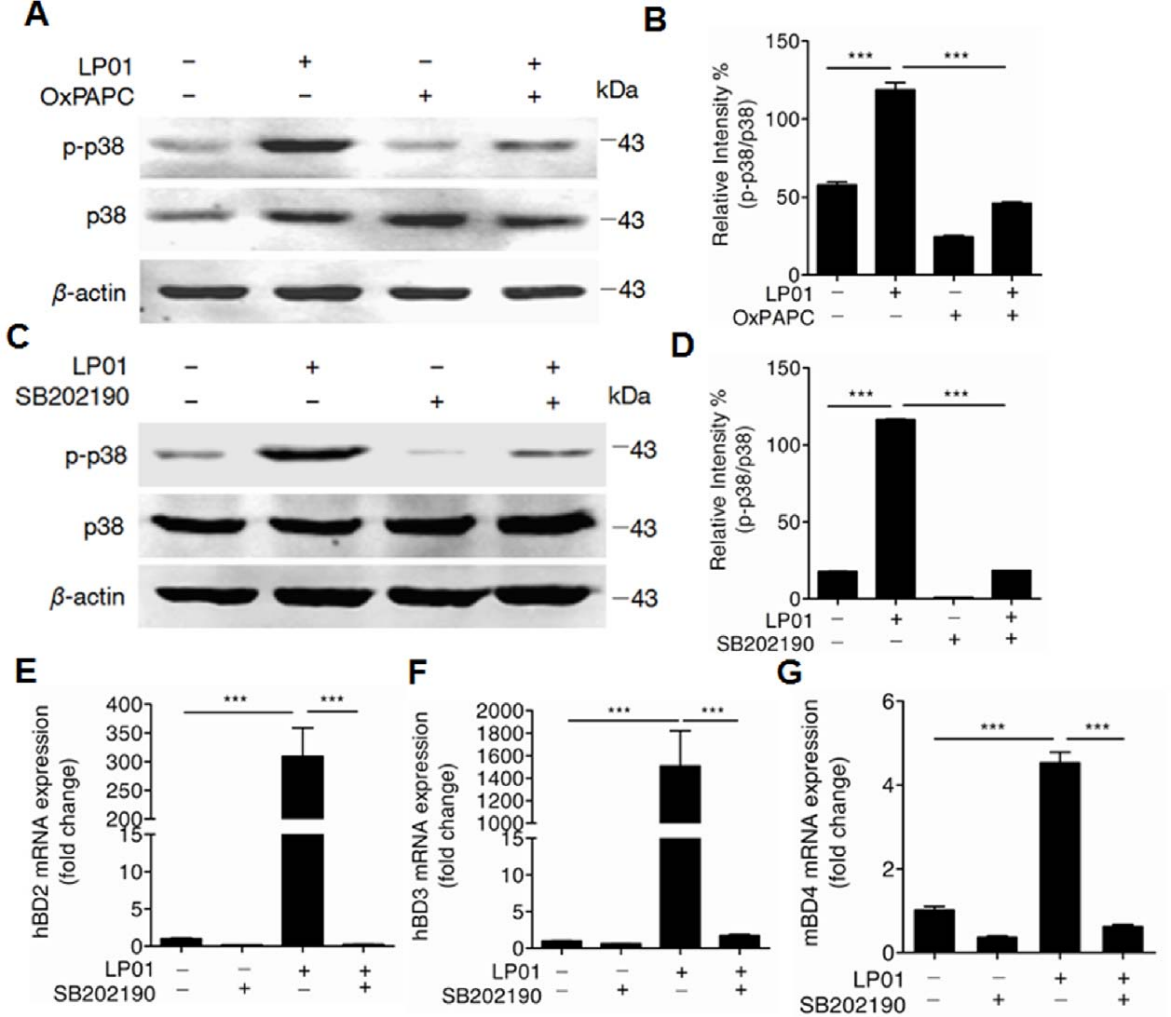
The activation of p38 MAPK is required for the induction of beta-defensins by LP01. **A&B.** TLR2 inhibitor OxPAPC inhibited p38 phosphorylation induced by 15 µg/mL of LP01 in NHEK cells. **C&D.** p38 MAPK inhibitor SB202190 inhibited p38 phosphorylation induced by 15 µg/mL of LP01 in NHEK cells. **E&F.** p38 MAPK inhibitor SB202190 completely inhibited hBD2 and hBD3 expression induced by 15 µg/mL of LP01 in NHEK cells. **G.** p38 MAPK inhibitor SB202190 completely inhibited mBD4 expression induced by 15 µg/mL of LP01 in murine primary keratinocytes. ***P<0.001. *P* values were determined by one-way ANOVA. Data are the means ± SEM of *n* = 3 and representative of two to three independent experiments.

It has been reported that activation of TLR2 regulates multiple downstream molecules including NF-κB and mitogen-activated protein kinases(MAPKs) [15]. Specifically, p38 MAPK has been shown to play an important role in hBD2 and hBD3 production in epithelial cells such as keratinocytes [16], [17]. We thus hypothesized that p38 MAPK might be the downstream molecule of TLR2 involved in the induction of hBDs by LP01. To test this hypothesis, we first checked if LP01 induced p38 MAPK phosphorylation after TLR2 was blocked. TLR2 inhibitor OxPAPC markedly decreased the phosphorylation of p38MAPK (Thr180/Tyr182) by LP01 (Fig. 6A and 6B). In addition to OxPAPC, p38 MAPK inhibitor, SB202190 completely inhibited p38 MAPK phosphorylation (Fig. 6C and 6D). Furthermore, the inhibition of p38 MAPK significantly decreased the expression of hBD2 and hBD3 in NHEKs (Fig. 6E and 6F) as well as mBD4 in primary murine keratinocytes (Fig. 6G). Taken together, these results demonstrate that p38 MAPK is the critical downstream molecule of TLR2 to induce hBD2 and hBD3 expression induced by LP01.

## Discussion

To date, most studies on skin microbes have been conducted to analyze the type and diversity of microbes present on the skin by classic culture techniques or identified by sequencing surveys of 16s rRNA. However, sparse attention has been paid to the beneficial function of these microbes that inhabit on our skin. Here we have identified one new lipopeptide (LP01) from *S.epidermidis*, one of the most commonly isolated bacterial species from healthy human skin [18], [19], and show that the lipopeptide activates TLR2/CD36-p38 MAPK to induce hBD2 and hBD3 expression, thus enabling the skin to mount an enhanced antimicrobial defense against pathogenic infections. Our results reveal that skin commensals play an important role in host antimicrobial defense and suggest that the preservation of these commensals on skin may be an effective way to achieve maintenance of healthy ‘normal’ skin function.

Epidemiologic and clinical studies indicate that the increased incidence of autoimmune and allergic diseases in developed countries is associated with reduced microbial exposure and alteration of microbial communities in various body sites [20]. Although high-throughput metabolomics analyses have been used to identify core microbial communities linked to the onset of pathologies, there is still a big challenging to define a ‘normal healthy’ microbiota of skin at the functional level [21], [22], [23]. Moreover, the molecular mechanisms underlying microbe-host interactions that shape host immune functions remain largely unknown. Previous work from Schittek and our group has demonstrated that skin commensals induce antimicrobial peptide RNase7 to amplify the innate immune response [24] and that *S.epidermidis* conditioned culture medium (SECM) increases antimicrobial peptide expression in keratinocytes [5]. Here we further advance our understanding of how skin commensal *S.epidermidis* regulates host immune responses against bacterial infections by defining the structure of lipopeptide LP01 from *S.epidermidis* and delineating the mechanism by which LP01 induces the expression of antimicrobial peptide hBD2 and hBD3.

In order to identify the structure of LP01, we used Q-TOF mass spectrometry *de-novo* sequencing technique to identify the amino acid sequence of the peptide chain and gas chromatography mass spectrometry technique to identify the fatty acid chain as priviously described [25], [26]. According to the amino acid sequence of the lipopeptide, we found that there were two possiblities for fatty acid heneicosanoic acid binding to peptide chain. One possibility was that heneicosanoic acid bound to Lys^11,^ the other was that heneicosanoic acid bound to N-terminal amino acid Asp^1^. To determine structure-function relationship, the above two possible lipopeptides were synthesized and their capacity to induce hBD2 and hBD3 expression was tested. Our results demonostrate that only the lipopeptide with heneicosanoic acid binding to Lys^11^ has the capacity to induce hBD2 and hBD3 expression, suggesting that Lys^11^ is the binding site of heneicosanoic acid. Furthermore, we have synthesized several derivatives of LP01 to prove that the intact peptide chain is essensial for the function of LP01 to induce AMPs. Our data show that heneicosanoic acid binding to shortened peptide chain completely lost its capacity to induce hBD2&3 while shortened fatty acid chain binding to the intact peptide chain kept its capacity to induce AMPs. These data suggest that the lipopeptide only with a unique structure has the capacity to induce AMPs.

Toll-like receptors (TLRs) have been reported to play an important role in host defense. They can recognize pathogen-associated molecular patterns (PAMPs) from microbes and damge-associated molecular patterns (DAMPs) from hosts. TLR2, as one of TLR family members, ususally works as TLR2/TLR1 or TLR2/TLR6 heterodimer and recognize a broad range of ligands, especially ligands of cell-wall componenets from gram-positive bacteria such as lipoteichoic acid (LTA) as well as peptidoglycan and lipoproteins [27]. LP01, as a lipopeptide from *S.epidermidis*, binds to TLR2 and then activates p38 MAPK to induce hBD2 and hBD3 expression. Furthrmore, we have shown that the coreceptor CD36 is also involved in the induction of hBD2 and hBD3 by LP01, which is consistent with previous observation that CD36 facilitates TLR2 recognition [28]. However, although it is reported that both CD36 and CD14 are required for TLR2 in response to LTA [29], [30], [31], in our system the silencing of CD14 only slightly decreased hBD2 expression but not hBD3 while knockdown of CD36 significantly reduced the expression of both hBD2 and hBD3. One explanation of this phenomenon is that CD36 and CD14 participate in different TLR2 signaling complex as CD36 is specifically involved in TLR2/TLR6-mediated response but not TLR2/TLR1-mediated response [29] while CD14 usually enhances signaling in respose to TLR2/TLR1 ligand Pam3CysSK4 [32]. The other explanation is that CD14 usually facilitates TLR2 ligands binding to TLR2 and NF-κB activation [32]. However, our data show that the induction of hBD2 and hBD3 requires p38 MAPK activation but not NF-κB activation. This is probably the reason that silencing of CD14 does not decrease the induction of hBD2 and hBD3 by LP01. Altogether, these data suggest that LP01, as a lipopeptide, activates TLR2/TLR6 signaling other than TLR2/TLR1 signaling. However, further investigation is needed.

In conclusion, these findings suggest that skin resident commensals are necessary for our protection from infections. Specifically, we find that lipopeptide LP01 from *S.epidermidis* exerts its effect by enhancing antimicrobial defense via the activation of TLR2/CD36-p38 MAPK signaling. Furthermore, our results indicate that identification of specific molecules from skin commensals to understand its roles in shaping host immune response is not only of primary importance for human health, but will also lead to the development of more rational antibacterial approaches. Our results also emphasize disadvantages of indiscriminate use of topical and systemic antibiotics to treat skin infections.
